# Diabetic Ketoacidosis: An Adverse Reaction to Immunotherapy

**DOI:** 10.7759/cureus.10632

**Published:** 2020-09-24

**Authors:** Dinesh Keerty, Manoj Das, Julie Hallanger-Johnson, Elizabeth Haynes

**Affiliations:** 1 Internal and Hospital Medicine, Moffitt Cancer Center, Tampa, USA; 2 Internal Medicine / Nephrology, Geisinger Health System, Danville, USA; 3 Endocrinology, Moffitt Cancer Center, Tampa, USA

**Keywords:** severe diabetic ketoacidosis, immunotherapy-related adverse events

## Abstract

Immune checkpoint inhibitors (ICPIs), such as anti-programmed death receptor 1 (PD-1) and anti-cytotoxic T-lymphocyte-associated antigen 4 (CTLA-4), are being utilized in the treatment of many malignancies. Just like their benefits in increasing recurrence-free survival, they also have shown numerous side effects affecting various organ systems. The endocrine adverse events can range from diabetes, hypothyroidism to diabetic ketoacidosis, and adrenal crisis. We would like to report a case of diabetic ketoacidosis (DKA) secondary to combination ipilimumab and nivolumab therapy after two doses. A 49-year-old female presented to the emergency department with nausea and vomiting. Her labs revealed blood glucose of 384 mg/dL, positive ketones, glucose in the urine, and an arterial pH of 7.2. She was treated as per our diabetic ketoacidosis protocol and ultimately discharged on insulin therapy. Clinicians should be vigilant about new hyperglycemic episodes in their patients who are on immunotherapy. Timely detection and management lead to better outcomes. Insulin is the standard treatment of choice in the treatment of immunotherapy mediated type 1 diabetes mellitus.

## Introduction

Immune checkpoint inhibitors (ICPIs), such as anti-programmed death receptor 1 (PD-1) and anti-cytotoxic T-lymphocyte-associated antigen 4 (CTLA-4), are being utilized in the treatment of many malignancies [1]. Just like their benefits in increasing recurrence-free survival, they also have shown numerous side effects affecting various organ systems [2]. The endocrine adverse events can range from diabetes, hypothyroidism to diabetic ketoacidosis, and adrenal crisis [3]. We would like to report a case of diabetic ketoacidosis (DKA) secondary to combination ipilimumab and nivolumab therapy.

## Case presentation

A 49-year-old female with small cell lung cancer presented to the emergency department with nausea and vomiting. She endorsed difficulty tolerating oral food and liquids, including her medications. She has a strong history of superior vena cava syndrome for the past one year when she was diagnosed with cancer. She had undergone concurrent chemo-radiation over the past year. Due to progressive disease, she was started on immunotherapy with ipilimumab and nivolumab. She received two doses with the last one being one week ago. She had been experiencing intermittent, crampy abdominal pain, diffuse in nature, with no radiating features. She also complained of exertional dyspnea that was more acute than her usual. She has chronic shortness of breath due to her cancer over the past year but never needed oxygen. She was placed on a 2 L nasal cannula due to mild respiratory distress that was noted. She denied any episodes of hemoptysis, hematemesis, new substernal chest pain, or any other acute complaints. We obtained complete blood count, chemistry, urinalysis, and arterial blood gas (Table 1).

**Table 1:** Labs on presentation

Lab	Patient’s Value	Normal Range
CHEMISTRY		
Sodium	129 mmol/L	135–145 mmol/L
Potassium	3.8 mmol/L	3.3–5.0 mmol/L
Chloride	90 mmol/L	98–107 mmol/L
Total Bicarbonate	14 mmol/L	21–32 mmol/L
Glucose	384 mg/dL	65–110 mg/dL
Lipase	6 U/L	13-60 U/L
Blood Acetone	26 mg/dL	<5 mg/dL
Hemoglobin A1C	6.6%	4.8-5.9%
URINALYSIS		
Glucose	>500 mg/dL	None
Ketones	80 mg/dL	None
ARTERIAL BLOOD GAS		
pH	7.27	7.35-7.45
pCO2	35 mmHg	35-45 mmHg
HCO3	15 mEq/L	22-26 mEq/L

We noted that the patient's total bicarbonate level was severely low along with an elevated blood glucose level. Her urinalysis showed positive ketones and glucosuria. However, the patient had no history of diabetes mellitus. We initiated our hospital's diabetic ketoacidosis protocol. We gave her bolus normal saline, regular insulin, and started her on an insulin drip. She was maintained on the insulin drip for 24 hrs, with rates ranging from 0.16-2.5 units/hour, before transitioning to subcutaneous insulin therapy. Over the next four days, her glucose level ranged between 150 and 220 mg/dL. She was able to tolerate her food and was discharged on Glargine and Novolog. She was evaluated by endocrinology who ordered a c-peptide level, insulin level, and glutamic acid decarboxylase (GAD 65) antibodies before discharge. Her hemoglobin A1C was 6.6% on admission suggesting reasonable blood sugar controls until this hospitalization. On her two-week follow-up, her glucose levels were better controlled on her insulin regimen. The labs showed new development of type 1 diabetes mellitus (Table 2).

**Table 2:** Autoimmune diabetes panel GAD: Glutamic acid decarboxylase; ACTH: Adrenocorticotropic hormone; TSH: Thyroid-stimulating hormone.

Lab	Patient’s Value	Normal Range
C-peptide	<0.1 ng/mL	0.8–3.5 ng/ml
GAD antibodies	<5 IU/ml	<5 IU/ml
Insulin level	19 uIU/mL	3-19 uIU/mL
Insulin Antibody	<0.4 U/mL	Value greater than 0.4 U/mL is positive
Cortisol	8.4 ug/dL	6.2-23.3 ug/dL
ACTH	7.4 pg/mL	7.2-63.3 pg/mL
TSH	32.3 uIU/mL	0.27-4.20 uIU/mL

She continued to follow her insulin management and did not have any more recurrent episodes of diabetic ketoacidosis. She completed an additional two cycles of immunotherapy but ultimately died due to disease progression.

## Discussion

The destruction of insulin-producing β-cells by T lymphocytes leads to the development of Type 1 diabetes mellitus. Studies in mice with genetic deletions of CTLA-4 and PD-1 have shown systemic development of autoimmune diseases, such as Type 1 diabetes mellitus [3, 4]. Therefore, it can be surmised that anti-PD-1 drugs could lead to similar findings in humans [5]. In patients with type 1 diabetes mellitus, they have been noted to have a reduced PD-1 expression in CD4+ T cells, which might indicate that the expression in CD4+ T cells might contribute to the development of type 1 diabetes [6]. A systematic review and meta-analysis performed by Tzoulis et al. noted that patients in 38 randomized trials reported an overall incidence of about 10% for immunotherapy-related clinically significant endocrinopathies [7]. Clotman et al. noted the median onset of diabetes mellitus after immunotherapy was six weeks, median HbA1C was 7.5%, and about 56% showed glutamic acid decarboxylase activity, a prominent indicator of autoimmune diabetes [5]. Even Gauci et al. showed that approximately three-quarters of patients presented with diabetic ketoacidosis [8].

Most immunotherapy-related adverse events respond well when treated with high-dose corticosteroids [9]. However, it has not been effective in the treatment of autoimmune diabetes mellitus. Since Type 1 diabetes manifests when more than 80% of pancreatic β-cells have been destroyed, initiation of corticosteroids only led to poor glycemic control [5, 10-11]. Therefore, the current management of immunotherapy-induced diabetes remains standard insulin therapy.

Immunotherapy-induced hyperglycemia should be considered whenever there is a new onset fasting glucose > 200 mg/dL or random blood glucose > 250 mg/dL [12]. Clotman et al. also noted that insulin therapy led to glycemic control in all but three of the forty-two cases [5]. Once glycemic control was achieved, immunotherapy was restarted. Patients were then able to continue the utilization of immune checkpoint inhibitors for treatment. Based on the above, our case seems to be in line with autoimmune diabetes occurring after an event of diabetic ketoacidosis. This further implies the need for close monitoring for acute fulminant diabetes in patients on immunotherapy.

## Conclusions

In our patient, the diagnosis of diabetic ketoacidosis was made due to the presence of ketones in the urine, anion gap acidosis, and hyperglycemia. Our further laboratory evaluation showed absent c-peptide levels indicative of acute glycemic attack. The patient was treated for insulin-deficient or type 1 diabetes which resulted in overall improvement. The patient was able to maintain good glycemic control through the rest of her therapy. She ultimately died from disease progression within six months.

Clinicians should be vigilant about new hyperglycemic episodes in their patients who are on immunotherapy. Timely detection and management lead to better outcomes. Insulin is the standard treatment of choice in the treatment of immunotherapy-mediated type 1 diabetes mellitus.

## References

[1] Ugurel S, Kiecker F, Fröhling S (2017). Fulminant response to combined checkpoint inhibition with ipilimumab plus nivolumab after failure of nivolumab monotherapy in metastatic melanoma. Eur J Cancer.

[2] Trinh S, Le A, Gowani S, La-Beck NM (2019). Management of immune-related adverse events associated with immune checkpoint inhibitor therapy: a minireview of current clinical guidelines. Asia Pac J Oncol Nurs.

[3] Stamatouli AM, Quandt Z, Perdigoto AL (2018). Collateral damage: insulin-dependent diabetes induced with checkpoint inhibitors. Diabetes.

[4] Kavvoura FK, Ioannidis JP (2005). CTLA-4 gene polymorphisms and susceptibility to type 1 diabetes mellitus: a HuGE review and meta-analysis. Am J Epidemiol.

[5] Clotman K, Janssens K, Specenier P, Weets I, De Block CEM (2018). Programmed cell death-1 inhibitor-induced type 1 diabetes mellitus. J Clin Endocrinol Metab.

[6] Fujisawa R, Haseda F, Tsutsumi C (2015). Low programmed cell death-1 (PD-1) expression in peripheral CD4(+) T cells in Japanese patients with autoimmune type 1 diabetes. Clin Exp Immunol.

[7] Tzoulis P, Corbett RW, Ponnampalam S, Baker E, Heaton D, Doulgeraki T, Stebbing J (2018). Nivolumab-induced fulminant diabetic ketoacidosis followed by thyroiditis. Endocrinol Diabetes Metab Case Rep.

[8] Gauci ML, Laly P, Vidal-Trecan T (2017). Autoimmune diabetes induced by PD-1 inhibitor-retrospective analysis and pathogenesis: a case report and literature review. Cancer Immunol Immunother.

[9] Thompson J, Schneider B, Brahmer J (2019). Management of immunotherapy-related toxicities, version 1.2019. J Natl Compr Canc Netw.

[10] Aleksova J, Lau PK, Soldatos G, McArthur G (2016). Glucocorticoids did not reverse type 1 diabetes mellitus secondary to pembrolizumab in a patient with metastatic melanoma. BMJ Case Rep.

[11] Sakaguchi C, Ashida K, Yano S (2019). A case of nivolumab-induced acute-onset type 1 diabetes mellitus in melanoma. Curr Oncol.

[12] Barroso-Sousa R, Ott PA, Hodi FS, Kaiser UB, Tolaney SM, Min L (2018). Endocrine dysfunction induced by immune checkpoint inhibitors: practical recommendations for diagnosis and clinical management. Cancer.

